# Distinct modifiable risk factors and preventable burdens of preterm birth: a risk-stratified analysis of pregnancies with and without gestational diabetes mellitus

**DOI:** 10.3389/fped.2026.1725116

**Published:** 2026-02-24

**Authors:** Yuhang Wu, Lizhang Chen, Tingting Wang

**Affiliations:** 1 Department of Epidemiology and Health Statistics, Xiangya School of Public Health, Central South University, Changsha, Hunan, China; 2 Hunan Provincial Key Laboratory of Clinical Epidemiology, Xiangya School of Public Health, Central South University, Changsha, Hunan, China; 3 NHC Key Laboratory of Birth Defect for Research and Prevention, Hunan Provincial Maternal and Child Health Care Hospital, Changsha, Hunan, China

**Keywords:** epidemiology, gestational diabetes mellitus, population attributable fraction, preterm birth, risk factors

## Abstract

**Background:**

Preterm birth (PTB) remains a major clinical and public health challenge worldwide. Gestational diabetes mellitus (GDM), complicating 14%–25% of pregnancies, elevates PTB risk via metabolic dysregulation. Although various early-pregnancy exposures are associated with PTB, their differential contributions in GDM-affected and unaffected pregnancies remain inadequately explored. This study aimed to identify distinct first-trimester modifiable risk factors for PTB in these two populations and to quantify the accurately preventable burden using an advanced estimation approach that accounts for interdependencies among risk factors.

**Methods:**

In this prospective cohort study conducted in Central China (2019–2024), 2,825 pregnant women were stratified into GDM (*n* = 554) and non-GDM (*n* = 2,271) groups. Assessed early-pregnancy exposures included advanced maternal age, smoking, depressive symptoms, physical inactivity, insufficient sleep, and pre-pregnancy overweight or obesity. Multivariable logistic regression and principal component analysis-adjusted population attributable fractions (PAFs) were employed to estimate the preventable PTB proportion, adjusting for overlap among risk factors.

**Results:**

Six modifiable risk factors were identified for GDM pregnancies, with a combined PAF of 73.7% and an adjusted combined PAF of 50.5%. For non-GDM pregnancies, four factors yielded a combined PAF of 44.2% and an adjusted combined PAF of 21.5%. Shared significant factors included smoking (PAF 27.4%, adjusted PAF 11.7% in GDM vs. PAF 22.7%, adjusted PAF 9.1% in non-GDM), depressive symptoms (22.7%, 11.6% vs. 15.0%, 6.0%), and overweight or obesity (18.1%, 7.7% vs. 11.9%, 4.8%). Risk factors specific to GDM pregnancies were advanced maternal age (11.6%, 4.9%), physical inactivity (19.3%, 8.2%), and insufficient sleep (14.9%, 6.4%). Low education was uniquely associated with PTB in non-GDM pregnancies (3.7%, 1.5%).

**Conclusion:**

This study delineates distinct early-pregnancy modifiable risk profiles for PTB in GDM and non-GDM populations, supporting the development of targeted preventive strategies. Subsequent studies are warranted to validate these findings across diverse populations and to assess the effectiveness of tailored first-trimester interventions based on this risk stratification.

## 1 Introduction

Preterm birth (PTB), defined clinically as delivery before 37 completed weeks of gestation, represents a major public health issue worldwide. With an estimated annual incidence of 15 million infants and responsible for 11% of global live births, PTB remains a leading contributor to neonatal mortality and morbidity (1). This condition is linked to serious early-life complications such as respiratory distress syndrome, intraventricular hemorrhage, and necrotizing enterocolitis (2–4). Survivors of preterm birth face elevated risks of long-term health impairments, including compromised growth, neurodevelopmental disorders, and early-onset chronic diseases, with risks increasing as gestational age decreases and persisting after accounting for genetic liability (5–10). The consequences of PTB extend beyond health, imposing heavy economic burdens and social strains on families and healthcare infrastructures (11). This growing challenge highlights the pressing demand for evidence-informed approaches to reduce the impact of PTB.

Detecting modifiable risk factors in the first trimester is crucial for PTB prevention, as this period offers a vital opportunity for early intervention. Maternal obesity, insufficient micronutrient consumption, and psychosocial stress in early pregnancy have been linked to abnormal placental development and increased risk of preterm labor (12–14). Emerging evidence links these early exposures to downstream metabolic dysregulation, particularly gestational diabetes mellitus (GDM). Importantly, GDM is characterized by pregnancy-onset insulin resistance with inadequate β-cell compensation, creating a distinct metabolic and inflammatory milieu that can alter placental function and fetal development through pathways that differ from those in non-GDM pregnancies (15–17). This pathophysiological divergence supports viewing GDM not merely as a risk marker, but as a clinical state that can reshape the etiological architecture of adverse pregnancy outcomes. Under this framework, modifiable risk factors for PTB may operate through biological pathways that differ by GDM status, such as being exacerbated by the insulin resistance and chronic inflammation characteristic of GDM. Alternatively, their effect size may vary substantially between these populations (18–20). We therefore hypothesize that *a priori* stratification by GDM status is essential to uncover these distinct risk profiles. This approach, justified by established biological and clinical heterogeneity, aims to enable precision prevention strategies, which are currently hindered by a lack of GDM-stratified evidence. Recent advances in predictive modeling, exemplified by the GDM-specific risk stratification system developed by Cooray et al. for adverse pregnancy outcomes, underscore the potential of tailored interventions for high-risk subgroups (21). Nevertheless, evidence remains insufficient to justify PTB prevention strategies specifically tailored to GDM status.

The potential for PTB prevention is especially significant in China, which accounts for 10% of the global PTB burden. Population Attributable Fraction (PAF) analysis provides a powerful epidemiological tool for estimating the proportion of preventable cases through risk factor modification (22). A landmark population-based study by Bryce et al. found that 73% of spontaneous PTBs across 81 low- and middle-income countries could be attributed to 24 key risk factors (23). However, conventional PAF analyses often assume risk factor independence, an assumption frequently violated in practice. Factors such as sedentary lifestyle, obesity, and low socioeconomic status are closely correlated, potentially inflating combined PAF estimates and distorting the actual scope for prevention. This methodological shortcoming emphasizes the need for advanced analytical techniques that account for risk factor overlap and support intervention prioritization.

In response to these challenges, the present study aims to: (1) identify unique profiles of first-trimester modifiable risk factors for PTB among pregnant individuals with and without GDM, using a rigorously assembled prospective cohort; and (2) apply an advanced PAF estimation method that adjusts for interdependencies among risk factors to accurately quantify preventable PTB burden. By examining how GDM status modifies the relationship between early-pregnancy exposures and PTB, this research seeks to support the development of risk-stratified prevention guidelines and promote precision public health in maternal care. Given China's high birth volume and rising GDM prevalence, the results may help direct resources toward effective, subgroup-specific interventions and contribute to national maternal health policy improvements.

## 2 Materials and methods

### 2.1 Study design and participant enrollment

This prospective cohort study was carried out at Hunan Provincial Maternal and Child Health Care Hospital in Central China between September 2019 and June 2024. Eligible participants were pregnant women aged 18 years or older, who began prenatal care before 14 weeks of gestation and planned to continue care and deliver at the study hospital. Recruitment took place in outpatient departments covering reproductive medicine, obstetrics, and ultrasonography. All participants provided written informed consent at enrollment. Ethical approval was granted by the Ethics Committee of Xiangya School of Public Health, Central South University (approval no. XYGW-2019-020).

**Figure 1:**
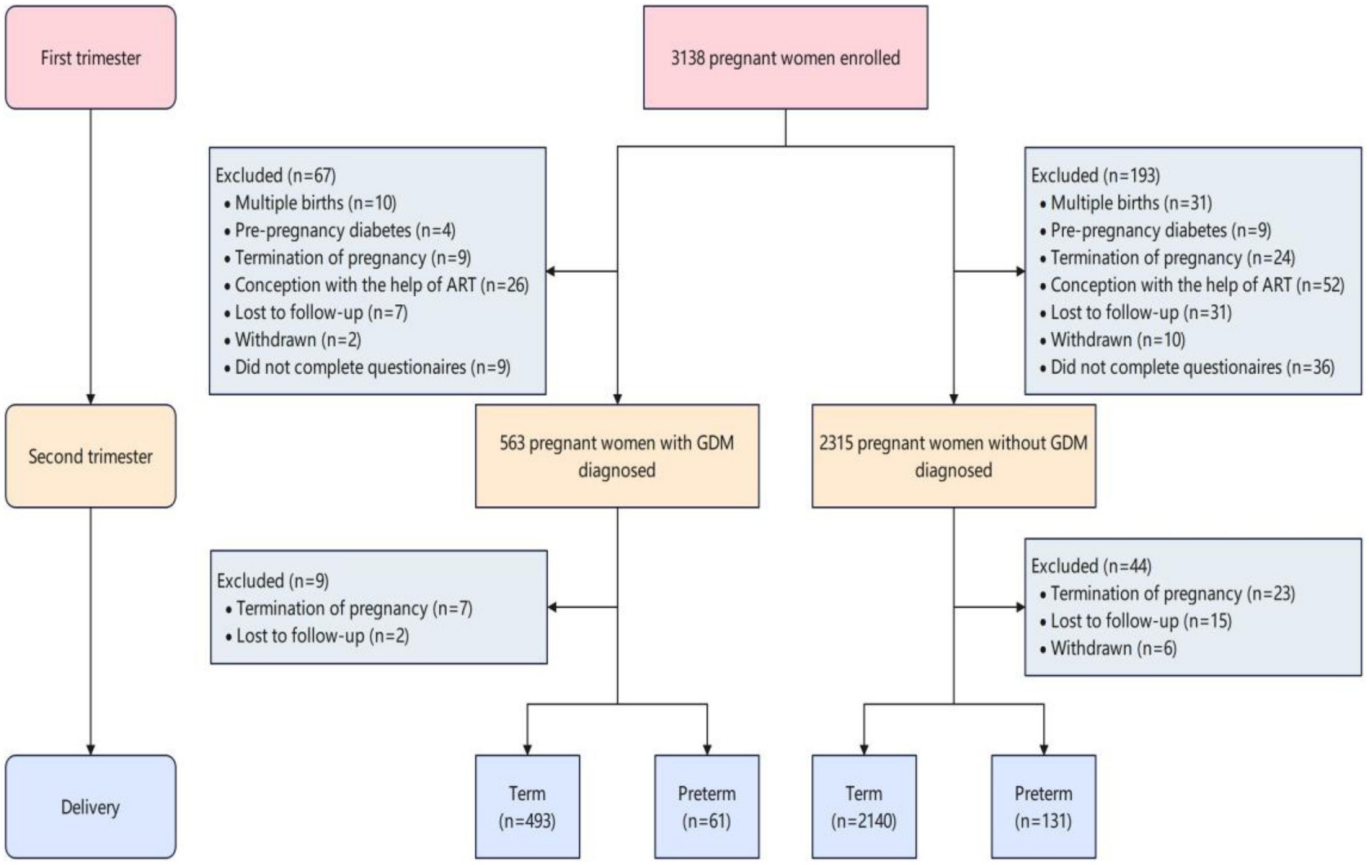
Flow chart showing study design and inclusion of participants.

Gestational age was determined using the first day of the last menstrual period (LMP) and verified via first-trimester ultrasound in cases of irregular cycles. To limit confounding, women who conceived using assisted reproductive technology (ART) were excluded (24). At 24–28 weeks of gestation, participants underwent a 75 g oral glucose tolerance test (OGTT) following International Association of Diabetes and Pregnancy Study Groups (IADPSG) criteria. Venous plasma glucose was measured at fasting, 1 h, and 2 h intervals using an automated analyzer (Toshiba TBA-120FR, Tokyo, Japan). GDM was diagnosed if any value met or exceeded the following thresholds: fasting ≥5.1 mmol/L, 1 h ≥10.0 mmol/L, or 2 h ≥8.5 mmol/L. Participants not meeting these criteria were classified as non-GDM. After applying all exclusion criteria, 2825 women remained in the final analysis (Figure 1).

### 2.2 Data collection

Trained interviewers collected baseline data using a structured questionnaire administered via face-to-face interviews and telephone follow-ups. Information on sociodemographic characteristics, behavioral habits, and lifestyle factors during early pregnancy (<14 weeks) was obtained. Questionnaire items and response options are summarized in Supplementary Figure S1. Variables included maternal age, educational attainment, smoking and alcohol use, physical activity levels, sleep duration, and mental health indicators. Self-reported data were cross-referenced with electronic medical records (EMRs) to ensure consistency. Data on pregnancy complications, clinical measures, and delivery outcomes were systematically retrieved from the hospital EMR system. Quality assurance procedures involved duplicate data entry and periodic audits by an independent team to resolve inconsistencies. Noteworthy, our analyses focused on modifiable exposures assessed in early pregnancy to estimate the early-prevention-attributable burden; time-varying clinical conditions typically ascertained later in gestation (e.g., gestational weight gain trajectories and hypertensive disorders of pregnancy) were not included in the primary models by design.

### 2.3 Exposures and covariates

Modifiable risk factors assessed in early pregnancy included: (1) advanced maternal age: defined as age ≥35 years at conception, verified using national identification records (24); (2) smoking: classified as active (self-reported inhalation of lit tobacco) or passive (exposure to secondhand smoke at least one day per week during early pregnancy) (25); (3) alcohol intake: consumption of any alcoholic beverage during early pregnancy, irrespective of amount (25); (4) overweight or obesity: body mass index (BMI) ≥24.0 kg/m², calculated from measured height and weight in lightweight clothing (26); (5) physical inactivity: low activity level according to the International Physical Activity Questionnaire-Short Form (IPAQ-SF), which classifies weekly activity as low, moderate, or high (27); (6) insufficient sleep: self-reported average sleep ≤7 h per day over the past week (28); (7) depressive symptoms: Edinburgh Postnatal Depression Scale (EPDS) score ≥10, validated for prenatal use in Chinese populations (29, 30); (8) low education: defined as senior middle school completion or less (28). Additional covariates included residence (urban/rural), parity (primiparous/multiparous), and ethnicity (Han/minority). Our analyses focused on modifiable exposures assessed in early pregnancy (<14 weeks) to estimate the early-prevention-attributable burden; time-varying clinical conditions typically ascertained later in gestation (e.g., gestational weight gain trajectories and hypertensive disorders of pregnancy) were not included in the primary models by design.

### 2.4 Outcomes

The primary outcome was PTB, defined as delivery occurring before 37 completed gestational weeks. Gestational age was ascertained using LMP and ultrasound data, with discrepancies resolved by prioritizing first-trimester ultrasound estimates. Delivery and neonatal outcomes were extracted from EMRs and validated by obstetricians blinded to exposure status.

### 2.5 Statistical analysis

Data entry and management were performed using EpiData software (version 3.1). A double-entry verification process was used to ensure accuracy, with discrepancies resolved by reviewing source documents. Participants were stratified by GDM status. Continuous variables following normal distributions are summarized as mean ± standard deviation (SD), and categorical variables as frequencies and percentages. Group comparisons used two-sample *t*-tests for continuous variables and Pearson's *χ*² or Fisher's exact tests for categorical variables.

Multivariable logistic regression was used to estimate odds ratios (ORs) and 95% confidence intervals (CIs) for associations between early-pregnancy modifiable risk factors and PTB, stratified by GDM status. To enhance interpretability and align with a prevention-oriented framework, we adopted a two-stage modeling strategy. Model 1 adjusted for core sociodemographic and obstetric covariates (residence, parity, ethnicity) selected *a priori* as potential confounders. Model 2 further included the set of candidate modifiable factors to estimate their independent associations with PTB and to support preventable burden estimation. We assessed multicollinearity using variance inflation factors (VIF) and Condition index. All VIF values were <2.5, indicating no problematic multicollinearity (Supplementary Tables S1, S2). Furthermore, Adjusted ORs from Model 2 were used to compute population attributable fractions (PAFs) for each factor using Levin's formula:

$$PAF=\frac{P\times (OR-1)}{1+P\times (OR-1)}$$

where *P* denotes the population prevalence of the risk factor and OR represents the corresponding odds ratio. The combined PAF under the assumption of independent risk factors was calculated as:

$$PAF=1-[(1-PAF_{1})(1-PAF_{2})(1-PAF_{3})\ldots ]$$

Because Levin's formula tends to overestimate combined PAFs in the presence of correlated exposures (23), we implemented a weighted PAF approach based on Principal Component Analysis (PCA). Specifically, PCA was conducted separately within the GDM and non-GDM strata and was restricted to potentially preventable exposures, rather than all study variables, to reflect the correlation structure among candidate intervention targets. A tetrachoric correlation matrix was constructed to capture pairwise associations among binary risk factors. PCA was applied to this matrix to extract eigenvalues and factor loadings, and the number of components retained was determined using parallel analysis. Communality for each risk factor, defined as the proportion of variance explained by shared latent factors, was computed as the sum of squared loadings across retained components. Detailed PCA results are provided in the Supplementary Tables S3–S5 and Supplementary Figure S2. Each PAF was then weighted as follows:

$$PAF=1-[(1-w_{1}\times PAF_{1})(1-w_{2}\times PAF_{2})(1-w_{3}\times PAF_{3})\ldots ]$$

where the $w_{i}$ was 1 minus the communality of each risk factor. This downweighs PAFs for factors with high shared variance, reflecting their reduced unique contribution to PTB risk. Final adjusted combined PAFs were obtained by summing weighted individual PAFs. Further methodological details are available in prior publications (22, 31).

All statistical analyses and visualizations were performed using R (version 4.2.0). A two-sided *P* value < 0.05 was considered statistically significant.

## 3 Results

The analytical sample consisted of 2,825 participants enrolled during the first trimester, including 554 diagnosed with GDM and 2,271 without GDM (Figure 1). A total of 192 preterm births (6.8%) were recorded. Baseline characteristics differed significantly between PTB cases occurring in GDM and non-GDM pregnancies (Supplementary Table S1). Participants with GDM and PTB were older at delivery (mean age 31.23 vs. 28.99 years), had higher pre-pregnancy BMI (21.84 vs. 20.74 kg/m²), and showed greater prevalence of advanced maternal age (26.2% vs. 8.4%), alcohol intake (14.8% vs. 5.3%), and depressive symptoms (60.7% vs. 44.3%). By contrast, primiparity was more common among non-GDM pregnancies with PTB than among their GDM-affected counterparts.

When comparing GDM pregnancies with and without PTB (Table 1), those ending in PTB had higher rates of advanced maternal age (26.2% vs. 10.5%), overweight or obesity (37.7% vs. 16.8%), smoking (54.1% vs. 35.1%), physical inactivity (39.3% vs. 20.7%), insufficient sleep (26.2% vs. 12.4%), and depressive symptoms (60.7% vs. 43.2%). Among non-GDM pregnancies (Table 2), PTB cases were more likely to reside in rural areas (88.6% vs. 80.9%), smoke (53.4% vs. 37.5%), have overweight or obesity (26.7% vs. 16.4%), exhibit depressive symptoms (44.3% vs. 31.8%), and have low educational attainment (21.4% vs. 12.0%) compared to term deliveries.

**Table 1:** Characteristics of the participants with gestational diabetes mellitus with or without preterm birth.

Characteristic	PTD (*N* = 61)	Non-PTD (*N* = 493)	All (*N* = 554)
Age at delivery (years)**	31.23 ± 4.20	30.47 ± 3.95	30.56 ± 3.98
Advanced maternal age (≥35 years)**			
Yes	16 (26.2)	52 (10.5)	68 (12.3)
No	45 (73.8)	441 (89.5)	486 (87.7)
Residence			
Urban	54 (88.5)	448 (90.9)	502 (90.6)
Rural	7 (11.5)	45 (9.1)	52 (9.4)
Pre-pregnancy BMI (kg/m^2^)	21.84 ± 3.55	21.38 ± 3.12	21.43 ± 3.17
Overweight or obese (BMI ≥ 24.0 kg/m^2^)**			
Yes	23 (37.7)	83 (16.8)	106 (19.1)
No	38 (62.3)	410 (83.2)	448 (80.9)
Parity			
Primiparous	33 (54.1)	289 (58.6)	322 (58.1)
Multiparous	28 (45.9)	204 (41.4)	232 (41.9)
Ethnicity			
Han	57 (93.4)	467 (94.7)	524 (94.6)
Minority	4 (6.6)	26 (5.3)	30 (5.4)
Education level			
Senior middle school or below	9 (14.8)	47 (9.5)	56 (10.1)
College or above	52 (85.2)	446 (90.5)	498 (89.9)
Alcohol intake			
Yes	9 (14.8)	47 (9.5)	56 (10.1)
No	52 (85.2)	446 (90.5)	498 (89.9)
Smoking**			
Yes	33 (54.1)	173 (35.1)	206 (37.2)
No	28 (45.9)	320 (64.9)	348 (62.8)
Physical inactivity**			
Yes	24 (39.3)	102 (20.7)	126 (22.7)
No	37 (60.7)	391 (79.3)	428 (77.3)
sleep duration (hours)**			
≤7.0	16 (26.2)	61 (12.4)	77 (13.9)
>7.0	45 (73.8)	432 (87.6)	477 (86.1)
Depressive symptoms**			
Yes	37 (60.7)	213 (43.2)	250 (45.1)
No	24 (39.3)	280 (56.8)	304 (54.9)

Continuous data are presented as mean ± SD, and categorical data are presented as *n* (%).

Two-sample *t* tests were used to compare mean values, and Pearson's Chi-square or Fisher's exact test was used to test the difference between proportions.

PTD, preterm birth; BMI, body mass index.

** *P* < 0.01.

**Table 2:** Characteristics of the participants without gestational diabetes mellitus with or without preterm birth.

Characteristic	PTD (*N* = 131)	Non-PTD (*N* = 2,140)	All (*N* = 2,271)
Age at delivery (years)	28.99 ± 4.13	29.20 ± 3.94	29.19 ± 3.95
Advanced maternal age (≥35 years)			
Yes	11 (8.4)	129 (6.0)	140 (6.2)
No	120 (91.6)	2,011 (94.0)	2,131 (93.8)
Residence**			
Urban	106 (80.9)	1,895 (88.6)	2,001 (88.1)
Rural	25 (19.1)	245 (11.4)	270 (11.9)
Pre-pregnancy BMI (kg/m^2^)	20.74 ± 2.69	20.41 ± 2.76	20.43 ± 2.75
Overweight or obese (BMI ≥ 24.0 kg/m^2^)**			
Yes	35 (26.7)	351 (16.4)	386 (17.0)
No	96 (73.3)	1,789 (83.6)	1,885 (83.0)
Parity			
Primiparous	92 (70.2)	1,441 (67.3)	1,533 (67.5)
Multiparous	39 (29.8)	699 (32.7)	738 (32.5)
Ethnicity			
Han	120 (91.6)	2,016 (94.2)	2,036 (94.1)
Minority	11 (8.4)	124 (5.8)	135 (5.9)
Education level**			
Senior middle school or below	28 (21.4)	256 (12.0)	284 (12.5)
College or above	103 (78.6)	1,884 (88.0)	1,987 (87.5)
Alcohol intake			
Yes	7 (5.3)	135 (6.3)	142 (6.3)
No	124 (94.7)	2,005 (93.7)	2,129 (93.7)
Smoking**			
Yes	70 (53.4)	802 (37.5)	872 (38.4)
No	61 (46.6)	1,338 (62.5)	1,399 (61.6)
Physical inactivity			
Yes	39 (29.8)	600 (28.0)	639 (28.1)
No	92 (70.9)	1,540 (72.0)	1,632 (71.9)
sleep duration (hours)			
≤7.0	21 (16.0)	270 (12.6)	291 (12.8)
>7.0	110 (84.0)	1,870 (87.4)	1,980 (87.2)
Depressive symptoms**			
Yes	58 (44.3)	681 (31.8)	739 (32.5)
No	73 (55.7)	1,459 (68.2)	1,532 (67.5)

Continuous data are presented as mean ± SD, and categorical data are presented as *n* (%).

Two-sample *t* tests were used to compare mean values, and Pearson's Chi-square or Fisher's exact test was used to test the difference between proportions.

PTD, preterm birth; BMI, body mass index.

** *P* < 0.01.

Multivariable logistic regression, adjusting for residence, parity, and ethnicity, identified distinct risk patterns between the two groups (Table 3). In the GDM cohort, advanced maternal age (OR 3.12, 95% CI 1.54–6.31), physical inactivity (OR 2.06, 95% CI 1.08–3.90), and insufficient sleep (OR 2.26, 95% CI 1.15–4.45) were significantly associated with PTB. In non-GDM pregnancies, low educational attainment was uniquely associated with PTB risk (OR 1.75, 95% CI 1.10–2.77). Risk factors common to both groups included smoking (GDM: OR 2.01, 95% CI 1.14–3.54; non-GDM: OR 1.76, 95% CI 1.22–2.54), overweight or obesity (GDM: OR 2.16, 95% CI 1.11–4.19; non-GDM: OR 1.79, 95% CI 1.19–2.71), and depressive symptoms (GDM: OR 1.83, 95% CI 1.03–3.26; non-GDM: OR 1.54, 95% CI 1.07–2.22).

**Table 3:** Related factors of preterm birth in patients with and without gestational diabetes mellitus based on multivariable logistic regression.

Characteristic	GDM		Non-GDM	
	Model 1	Model 2	Model 1	Model 2
Advanced maternal age	3.00 (1.55, 5.80)**	3.12 (1.54, 6.31)**	1.47 (0.77, 2.83)	1.36 (0.70, 2.65)
Smoking	2.18 (1.27, 3.73)**	2.01 (1.14, 3.54)*	1.85 (1.30, 2.65)**	1.76 (1.22, 2.54)**
Overweight or obese	2.97 (1.68, 5.25)**	2.16 (1.11, 4.19)*	1.88 (1.26, 2.83)**	1.79 (1.19, 2.71)**
Physical inactivity	2.50 (1.43, 4.37)**	2.06 (1.08, 3.90)*	1.08 (0.73, 1.59)	1.06 (0.71, 1.57)
Insufficient sleep	2.49 (1.33, 4.69)**	2.26 (1.15, 4.45)*	1.33 (0.82, 2.16)	1.16 (0.70, 1.90)
Depressive symptoms	2.04 (1.18, 3.52)*	1.83 (1.03, 3.26)*	1.67 (1.17, 2.39)**	1.54 (1.07, 2.22)*
Low education	1.58 (0.72, 3.46)	1.77 (0.77, 4.05)	1.99 (1.26, 3.13)**	1.75 (1.10, 2.77)*

Model 1 was adjusted for residence, parity, and ethnicity.

Medel 2 was adjusted for all potential exposures in addition to covariates in model A.

Two-sample *t* tests were used to compare mean values and Pearson's *χ*2 test was used to test the difference between proportions.

Values are presented as adjusted odds ratio with 95% confidence interval.

GDM, gestational diabetes mellitus.

* *P* < 0.05.

** *P* < 0.01.

**Figure 2:**
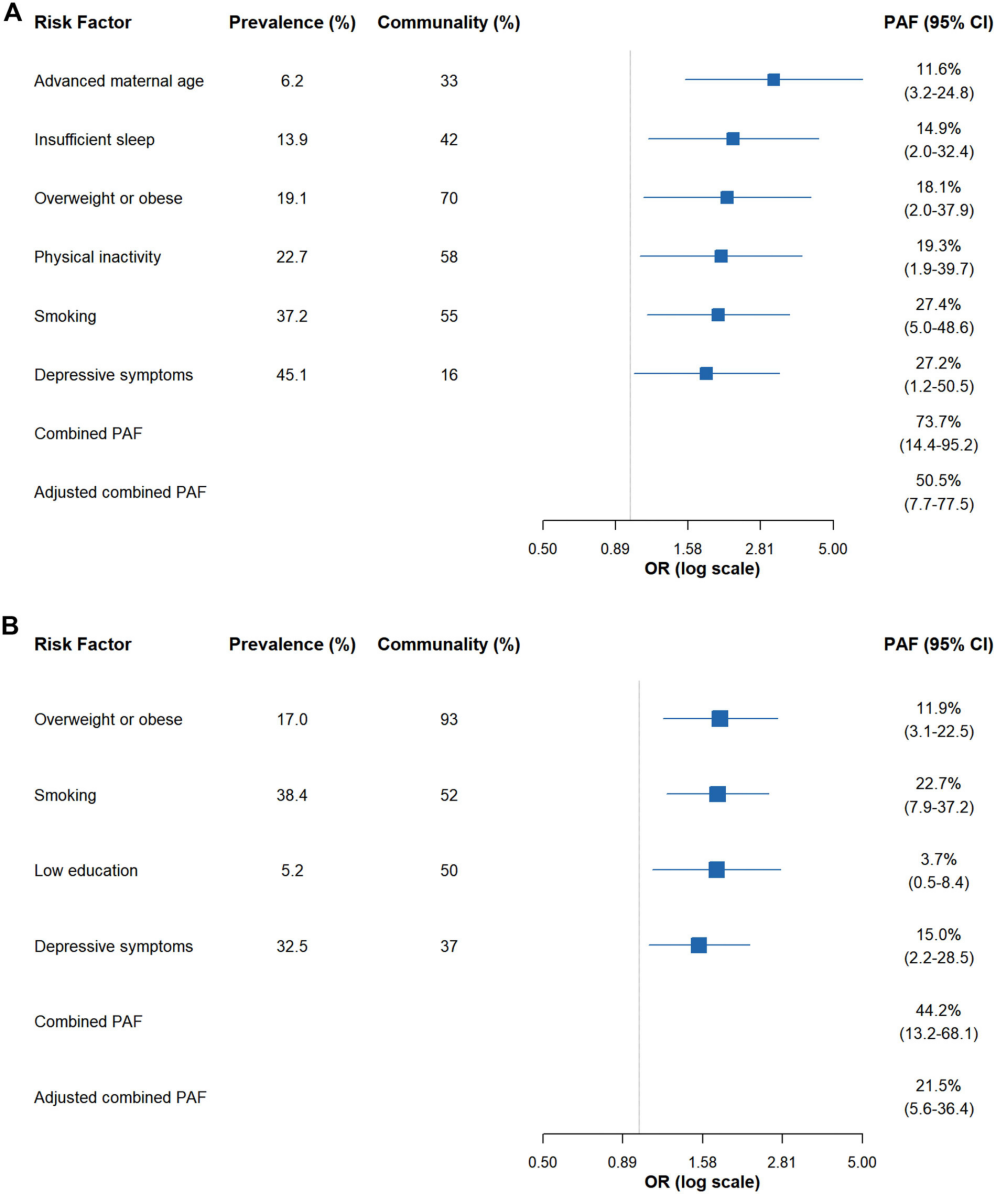
Estimates for population attribution fractions for preterm birth risk factors in participants with gestational diabetes mellitus **(A)** and non-gestational diabetes mellitus **(B)**.

**Figure 3:**
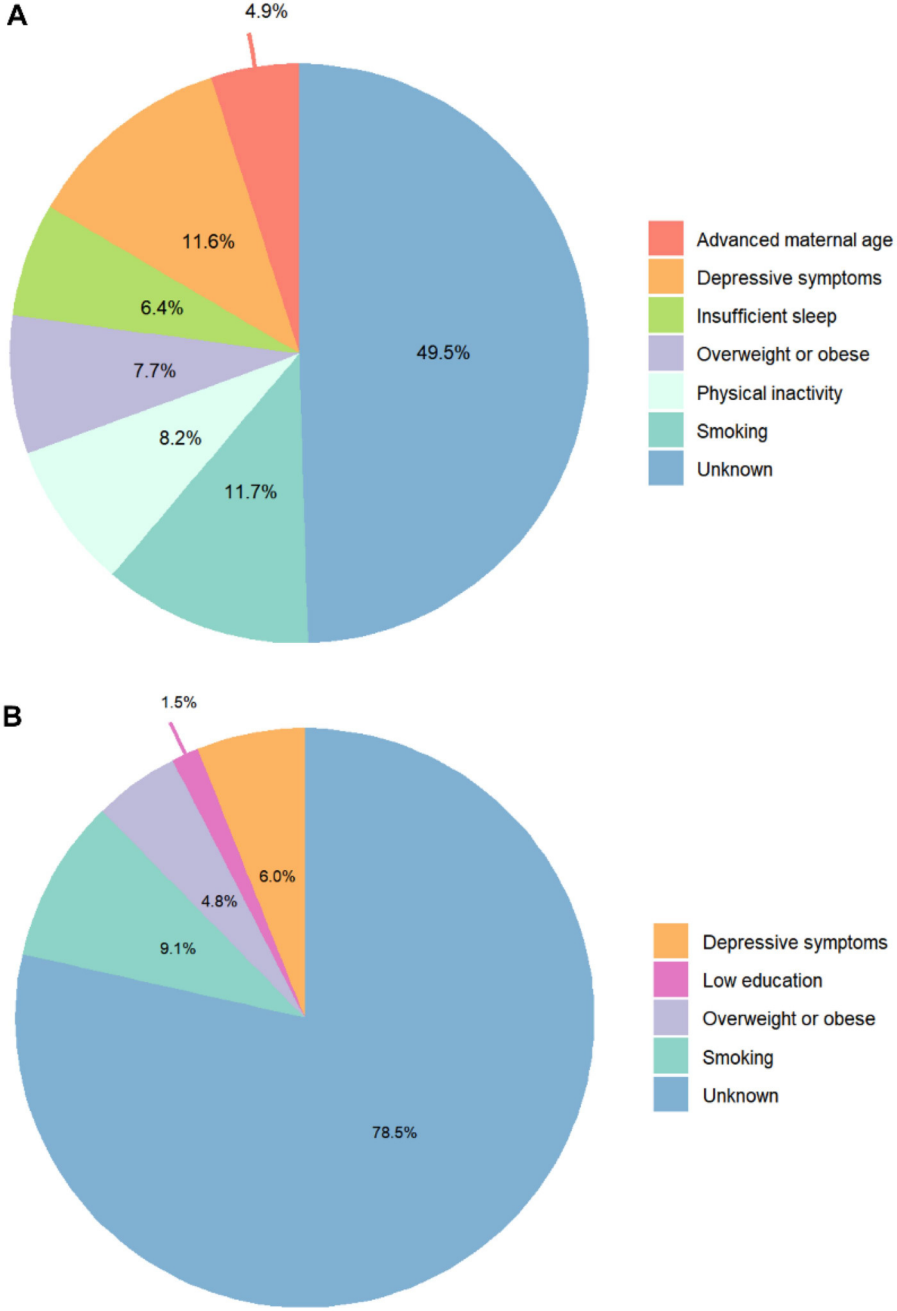
The adjusted population attribution fractions of risk factors for preterm birth. **(A)** Gestational diabetes mellitus. **(B)** Non-gestational diabetes mellitus.

Figure 2 summarizes the PAFs of modifiable risk factors for PTB in both groups. Among GDM pregnancies, six identified risk factors collectively accounted for a theoretical preventable proportion of 73.7% (95% CI 14.4–95.2). After adjusting for overlap between factors, the combined PAF decreased to 50.5% (95% CI 7.7–77.5). In non-GDM pregnancies, four risk factors yielded a combined PAF of 44.2% (95% CI 13.2–68.1) and an adjusted combined PAF of 21.5% (95% CI 5.6–36.4). Within the GDM group, smoking and depressive symptoms contributed most substantially to PTB burden, with unadjusted PAFs of 27.4% (95% CI 5.0–48.6) and 27.2% (95% CI 1.2–50.5), respectively. These were followed by physical inactivity (19.3%, 95% CI 1.9–39.7), overweight or obesity (18.1%, 95% CI 2.0–37.9), insufficient sleep (14.9%, 95% CI 2.0–37.9), and advanced maternal age (11.6%, 95% CI 3.2–24.8). After weighting for shared variance, smoking remained the leading contributor (adjusted PAF 11.7%), while advanced maternal age showed the lowest adjusted effect (4.9%) among the six factors (Figure 3A). In non-GDM pregnancies, smoking was also the predominant modifiable risk factor (PAF 22.7%, 95% CI 7.9–37.2), followed by depressive symptoms (15.0%, 95% CI 2.2–28.5), overweight or obesity (11.9%, 95% CI 3.1–22.5), and low education (3.7%, 95% CI 0.5–8.4). After adjustment, PAFs ranged from 1.5% for low education to 9.1% for smoking, indicating considerable attenuation of attributable risk after accounting for correlated exposures (Figure 3B).

## 4 Discussion

Our stratified analysis of modifiable first-trimester risk factors for PTB in pregnancies with and without gestational diabetes mellitus reveals important differences in preventable disease burden between these two populations. The identification of six significant risk factors in the GDM group vs. four in the non-GDM group suggests potentially divergent etiological pathways in the development of PTB. Using an advanced PAF approach that adjusts for overlapping risk pathways, we estimate that early-pregnancy interventions could prevent 50.5% of PTB cases in the GDM cohort and 21.5% in the non-GDM group. These results support a precision public health approach, emphasizing risk stratification to guide efficient resource deployment in perinatal care.

Unlike previous reports that estimated PAFs for PTB risk determinants (23), this study specifically assesses the preventive potential of modifiable lifestyle and behavioral factors acting during the first trimester. Smoking emerged as the leading modifiable risk factor in both groups (adjusted PAFs: 11.7% in GDM and 9.1% in non-GDM), consistent with existing evidence that prenatal tobacco exposure promotes placental dysfunction and systemic inflammatory responses—key pathways in PTB pathogenesis (32–34). Notably, more than half of the pregnant women in our cohort were exposed to active or passive smoking during early gestation, highlighting an urgent public health priority to incorporate smoking cessation support for pregnant individuals and their households into routine antenatal care. Depressive symptoms in early pregnancy also contributed considerably to PTB burden in both cohorts. One plausible mechanism involves stress-induced activation of the maternal hypothalamic-pituitary-adrenal axis, leading to elevated corticotropin-releasing hormone levels and predisposing to premature cervical changes (35). The higher PAF observed in GDM pregnancies may reflect a greater burden of undiagnosed mood disorders in this group, which is consistent with studies linking psychological distress to insulin resistance via glucocorticoid-mediated pathways (36). Overweight or obesity before pregnancy was consistently associated with PTB in both groups, though meta-analyses indicate heterogeneous effects across studies, possibly due to differing BMI cut-offs or population characteristics (37). While residual confounding cannot be ruled out, our findings are biologically plausible, as adiposity-related cytokines may contribute to chronic inflammation and impaired uteroplacental function (38, 39). Interaction analyses indicated that GDM status did not significantly modify the effect of these shared risk factors, supporting their general relevance in PTB prevention.

Several risk factors demonstrated group-specific effects. Advanced maternal age, physical inactivity, and insufficient sleep were independently associated with PTB only in the GDM population, suggesting that GDM-affected individuals may benefit from a broader set of preventive strategies. In China, where delayed childbearing is increasingly common, tailored metabolic health support for older pregnant women with GDM is especially warranted (40). The impact of physical inactivity reflects its dual pathological role in impairing insulin sensitivity while promoting pro-inflammatory cytokine production (41, 42). In our cohort, 8.2% of PTB cases in GDM pregnancies were attributable to inadequate physical activity, and nearly 40% of GDM participants were classified as inactive according to IPAQ-SF criteria, indicating a substantial opportunity for early intervention. Similarly, insufficient sleep may intensify inflammatory responses in GDM, exacerbating glucose intolerance and cervical maturation processes (43, 44). These observations contrast with Mendelian randomization studies that found no association between sleep duration and PTB (45), underscoring the importance of accounting for GDM-specific pathophysiology. In non-GDM pregnancies, low educational attainment was uniquely associated with PTB, possibly mediated by delayed initiation of prenatal care and limited health literacy, as observed in studies on socioeconomic disparities (46, 47). With ongoing educational expansion and quality improvements in China, the PTB burden attributable to low education is expected to decline.

Key strengths of this study include its prospective design and standardized screening procedures, which strengthen causal interpretation. Our results support the development of stratified PTB prevention guidelines, particularly within China's evolving maternal health system, especially under the three-child policy and rising GDM prevalence (48). Another important contribution is our application of an adjusted combined PAF method that addresses non-independence among risk factors. This is particularly relevant in real-world settings where modifiable exposures often cluster (e.g., depressive symptoms co-occurring with smoking), which can lead to double counting if PAFs are naively summed. Conventional PAF summation often yields estimates exceeding 100%, a statistical artifact resulting from violation of the mutual exclusivity assumption. As expected, our adjusted combined PAFs were lower than unadjusted estimates, reflecting more biologically plausible and stable measures of preventable burden (49). Both adjusted and unadjusted PAFs offer complementary insights: unadjusted values may better reflect the impact of eliminating a single risk factor, while adjusted estimates more realistically represent the effects of simultaneous multi-factorial interventions (50). Several limitations should be considered. First, key behavioral and lifestyle factors (e.g., sleep, physical activity, and smoking exposure) were primarily self-reported, which may introduce recall error and social desirability bias and potentially attenuate or inflate observed associations. Future studies could strengthen measurement validity by incorporating objective assessments, such as actigraphy for sleep and activity, and biomarker validation for tobacco exposure. In addition, depressive symptoms were captured via EPDS screening rather than clinical psychiatric diagnoses; residual confounding by unmeasured psychiatric history cannot be excluded and should be addressed in studies with structured diagnostic data. Second, this study was conducted in a single center in Central China; differences in population characteristics, healthcare access, and behavioral patterns across settings may limit generalizability. Multi-center replication and external validation across diverse regions are warranted. Third, although we propose plausible biological mechanisms, causal pathways remain incompletely elucidated. Future multi-omics studies using the biospecimen repository (maternal feces and serum) from this cohort may help address this gap. Finally, our PAF estimates are theoretical and require validation through intervention studies assessing actual risk reduction. These limitations, however, do not undermine the main conclusion that preventable PTB burdens differ meaningfully between GDM and non-GDM pregnancies, but instead highlight directions for refining risk quantification and mechanistic understanding.

## 5 Conclusion

This study enhances the framework of precision public health by delineating distinct profiles of modifiable first-trimester risk factors for PTB in pregnancies with and without GDM. Our findings provide robust evidence that GDM status significantly influences both the extent and composition of preventable PTB burden. The notably high preventable proportion among GDM pregnancies underscores the need for targeted antenatal initiatives that address modifiable metabolic and behavioral risk factors. Against the backdrop of China's increasing GDM prevalence and ongoing demographic transition, these results support the implementation of evidence-based, subgroup-specific strategies to reduce the population-level burden of preterm birth.

## Data Availability

The raw data supporting the conclusions of this article will be made available by the authors, without undue reservation.
