# Supplementary material for: Distinct modifiable risk factors and preventable burdens of preterm birth: a risk-stratified analysis of pregnancies with and without gestational diabetes mellitus
Source: Front Pediatr. 2026 Feb 24;14:1725116. doi: 10.3389/fped.2026.1725116 (PMC12971705; doi:10.3389/fped.2026.1725116)
Supplement: Supplementary file 1 [file Supplementaryfile1.docx]

**Supplementary Tables and Figures**

**Table S1 Collinearity statistics (Tolerance and VIF) for covariates, stratified by GDM status.**

| **Variable** | **Non-GDM Tolerance** | **Non-GDM**  **VIF** | **GDM**  **Tolerance** | **GDM**  **VIF** |
| --- | --- | --- | --- | --- |
| Physical inactivity | 0.989 | 1.011 | 0.942 | 1.061 |
| Overweight or obese | 0.990 | 1.011 | 0.893 | 1.120 |
| Insufficient sleep | 0.977 | 1.023 | 0.970 | 1.031 |
| Depressive symptoms | 0.975 | 1.026 | 0.976 | 1.025 |
| Smoking | 0.953 | 1.050 | 0.980 | 1.020 |
| Ethnicity | 0.993 | 1.007 | 0.988 | 1.013 |
| Parity | 0.949 | 1.054 | 0.935 | 1.070 |
| Residence | 0.980 | 1.020 | 0.973 | 1.028 |
| Advanced maternal age | 0.972 | 1.029 | 0.954 | 1.048 |
| Low education | 0.944 | 1.060 | 0.957 | 1.045 |
| Alcohol intake | 0.973 | 1.028 | 0.945 | 1.058 |

Note: Maximum VIF was 1.060 in the non-GDM stratum and 1.120 in the GDM stratum. GDM, Gestational diabetes mellitus; Non-GDM, Non-gestational diabetes mellitus.

**Table S2 Collinearity diagnostics (eigenvalues and condition indices) by dimension, stratified by GDM status.**

| **Dimension** | **Non-GDM Eigenvalue** | **Non-GDM Condition index** | **GDM**  **Eigenvalue** | **GDM Condition index** |
| --- | --- | --- | --- | --- |
| 1 | 5.185 | 1.000 | 5.465 | 1.000 |
| 2 | 1.004 | 2.273 | 1.034 | 2.299 |
| 3 | 0.923 | 2.370 | 0.958 | 2.388 |
| 4 | 0.915 | 2.380 | 0.870 | 2.506 |
| 5 | 0.814 | 2.524 | 0.789 | 2.632 |
| 6 | 0.792 | 2.559 | 0.782 | 2.643 |
| 7 | 0.656 | 2.812 | 0.558 | 3.131 |
| 8 | 0.627 | 2.876 | 0.543 | 3.174 |
| 9 | 0.557 | 3.051 | 0.502 | 3.298 |
| 10 | 0.444 | 3.416 | 0.427 | 3.576 |
| 11 | 0.064 | 8.985 | 0.054 | 10.023 |
| 12 | 0.019 | 16.557 | 0.017 | 17.963 |

Note: The maximum condition index was 16.557 (non-GDM) and 17.963 (GDM). GDM, Gestational diabetes mellitus; Non-GDM, Non-gestational diabetes mellitus.

**Table S3 PCA standardized loadings and communalities (h²) in the GDM group (nfactors = 2)**

| **Modifiable exposure** | **RC1**  **loading** | **RC2**  **loading** | **Communality (h²)** | **Uniqueness**  **(u²)** | **Complexity (com)** |
| --- | --- | --- | --- | --- | --- |
| Physical inactivity | 0.76 | -0.10 | 0.58 | 0.42 | 1.0 |
| Overweight or obese | 0.84 | 0.02 | 0.70 | 0.30 | 1.0 |
| Insufficient sleep | 0.47 | 0.45 | 0.42 | 0.58 | 2.0 |
| Depressive symptoms | 0.05 | 0.40 | 0.16 | 0.84 | 1.0 |
| Smoking | -0.01 | 0.74 | 0.55 | 0.45 | 1.0 |
| Advanced maternal age | -0.07 | 0.57 | 0.33 | 0.67 | 1.0 |

Note: Standardized loadings (pattern matrix) were obtained from PCA applied to the tetrachoric correlation matrix of binary modifiable exposures within the GDM stratum. Communality (h²) equals the sum of squared loadings across retained components; uniqueness u² = 1 − h². GDM, Gestational diabetes mellitus.

**Table S4 PCA standardized loadings and communalities (h²) in the non-GDM group (nfactors = 2)**

| **Modifiable exposure** | **RC1**  **loading** | **RC2**  **loading** | **Communality (h²)** | **Uniqueness**  **(u²)** | **Complexity (com)** |
| --- | --- | --- | --- | --- | --- |
| Overweight or obese | 0.00 | 0.97 | 0.93 | 0.068 | 1.0 |
| Depressive symptoms | 0.61 | -0.02 | 0.37 | 0.627 | 1.0 |
| Smoking | 0.71 | -0.12 | 0.52 | 0.483 | 1.1 |
| Low education | 0.65 | 0.28 | 0.50 | 0.499 | 1.4 |

Note: Standardized loadings (pattern matrix) were obtained from PCA applied to the tetrachoric correlation matrix of binary modifiable exposures within the GDM stratum. Communality (h²) equals the sum of squared loadings across retained components; uniqueness u² = 1 − h². Non-GDM, Non-gestational diabetes mellitus.

**Table S5 Component eigenvalues (SS loadings) and variance explained by retained components**

| **Group** | **Metric** | **RC1** | **RC2** | **Total / cumulative** |
| --- | --- | --- | --- | --- |
| GDM | SS loadings (eigenvalue) | 1.50 | 1.25 | 2.75 |
| GDM | Proportion of variance | 0.25 | 0.21 | 0.46 |
| GDM | Cumulative variance | 0.25 | 0.46 | 0.46 |
| GDM | Proportion explained | 0.54 | 0.46 | 1.00 |
| GDM | Cumulative proportion | 0.54 | 1.00 | 1.00 |
| Non-GDM | SS loadings (eigenvalue) | 1.30 | 1.02 | 2.32 |
| Non-GDM | Proportion of variance | 0.32 | 0.26 | 0.58 |
| Non-GDM | Cumulative variance | 0.32 | 0.58 | 0.58 |
| Non-GDM | Proportion explained | 0.56 | 0.44 | 1.00 |
| Non-GDM | Cumulative proportion | 0.56 | 1.00 | 1.00 |

Note: GDM, Gestational diabetes mellitus; Non-GDM, Non-gestational diabetes mellitus.


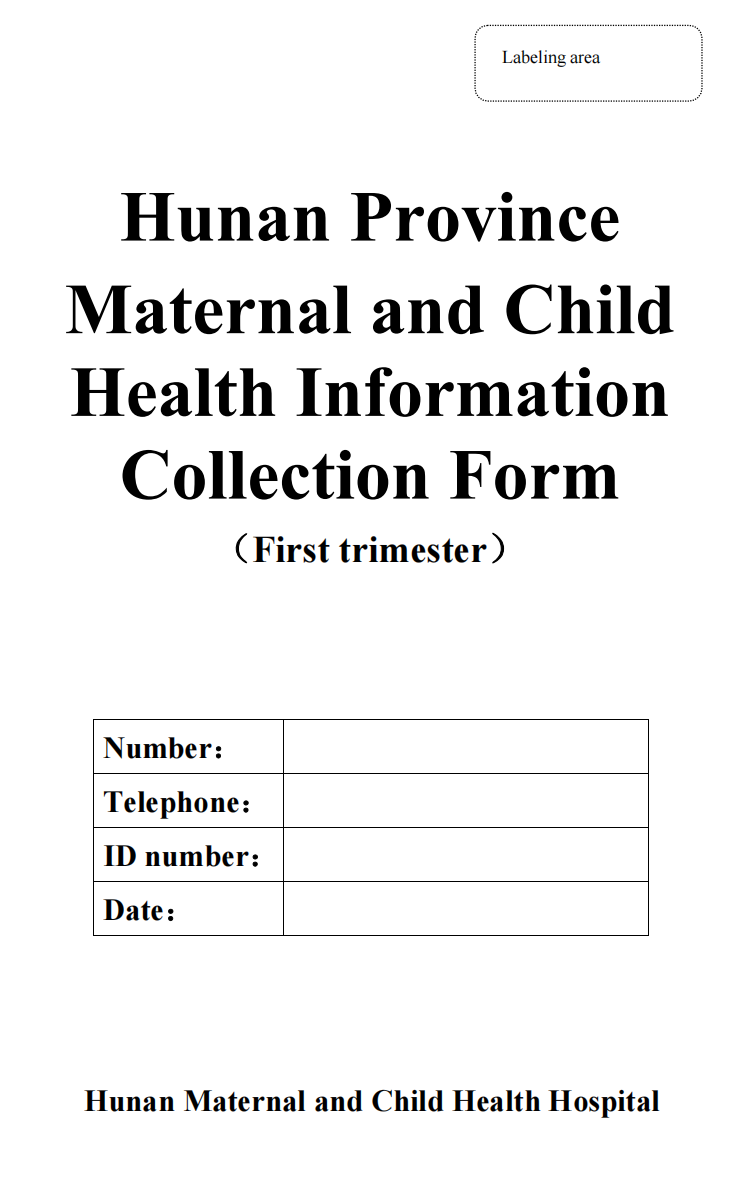


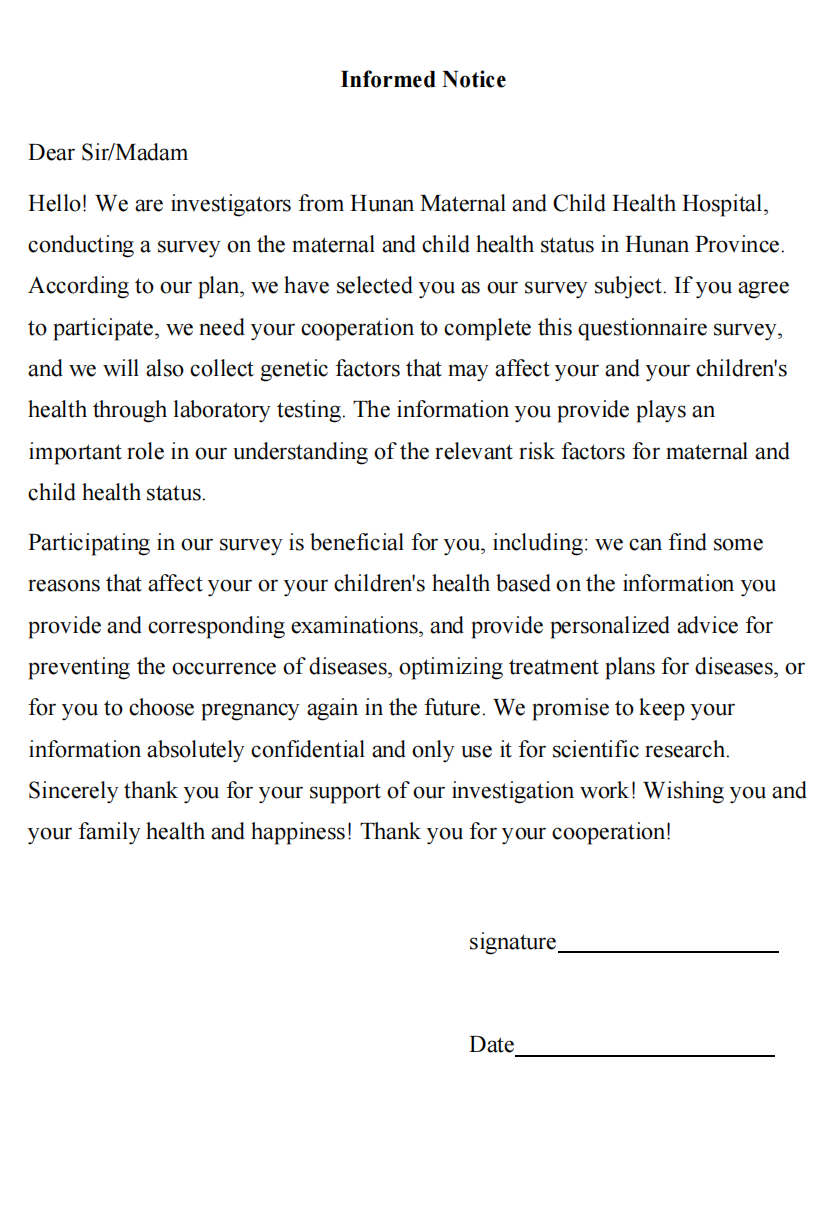


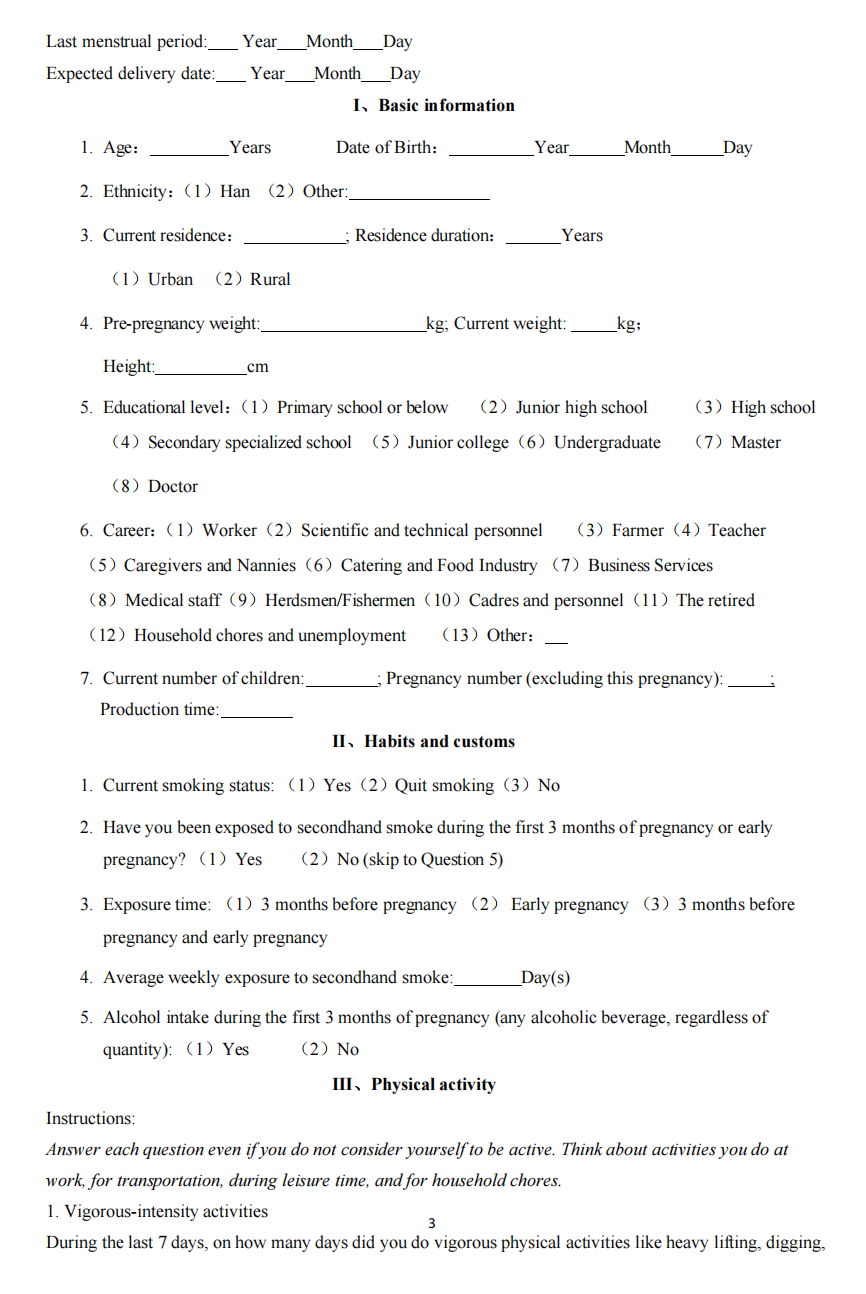


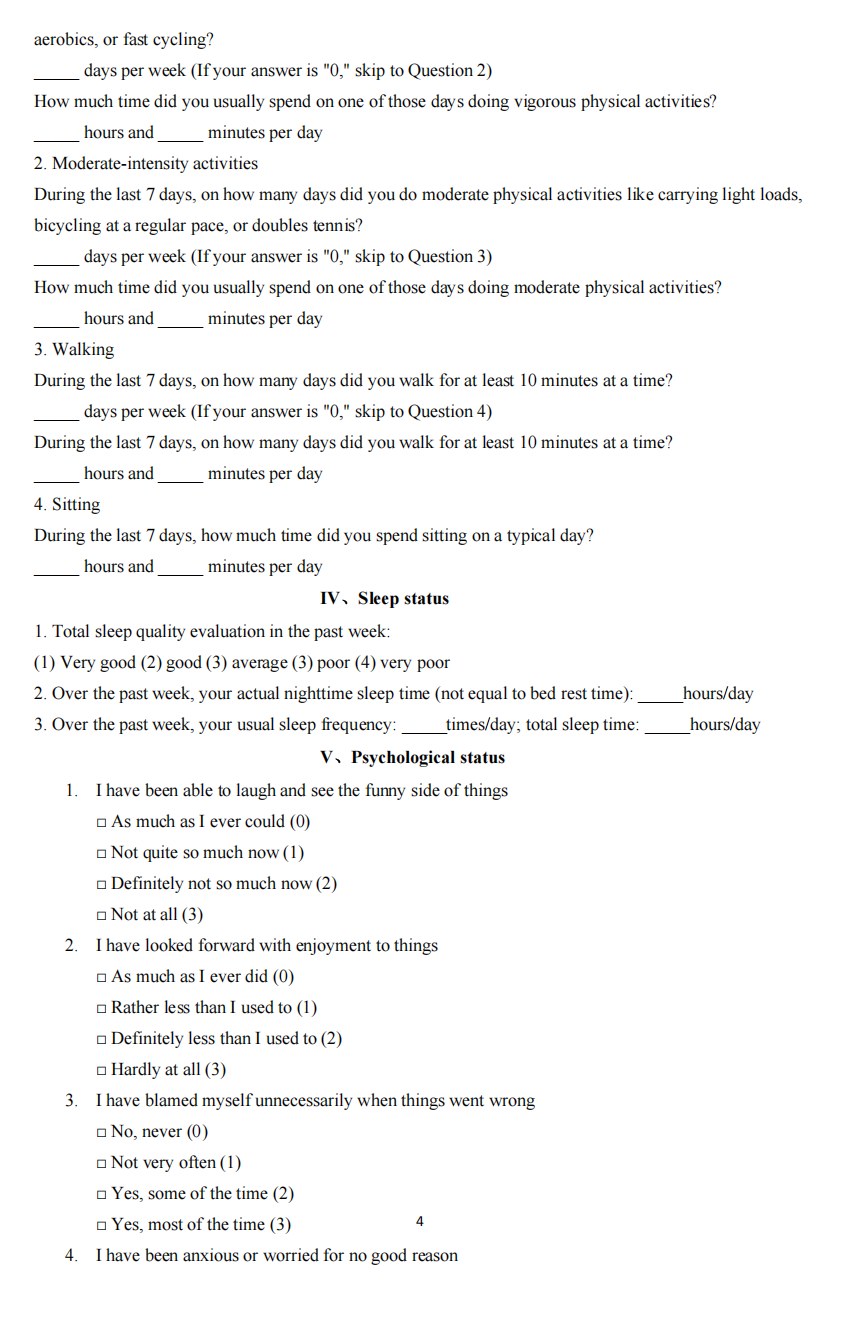


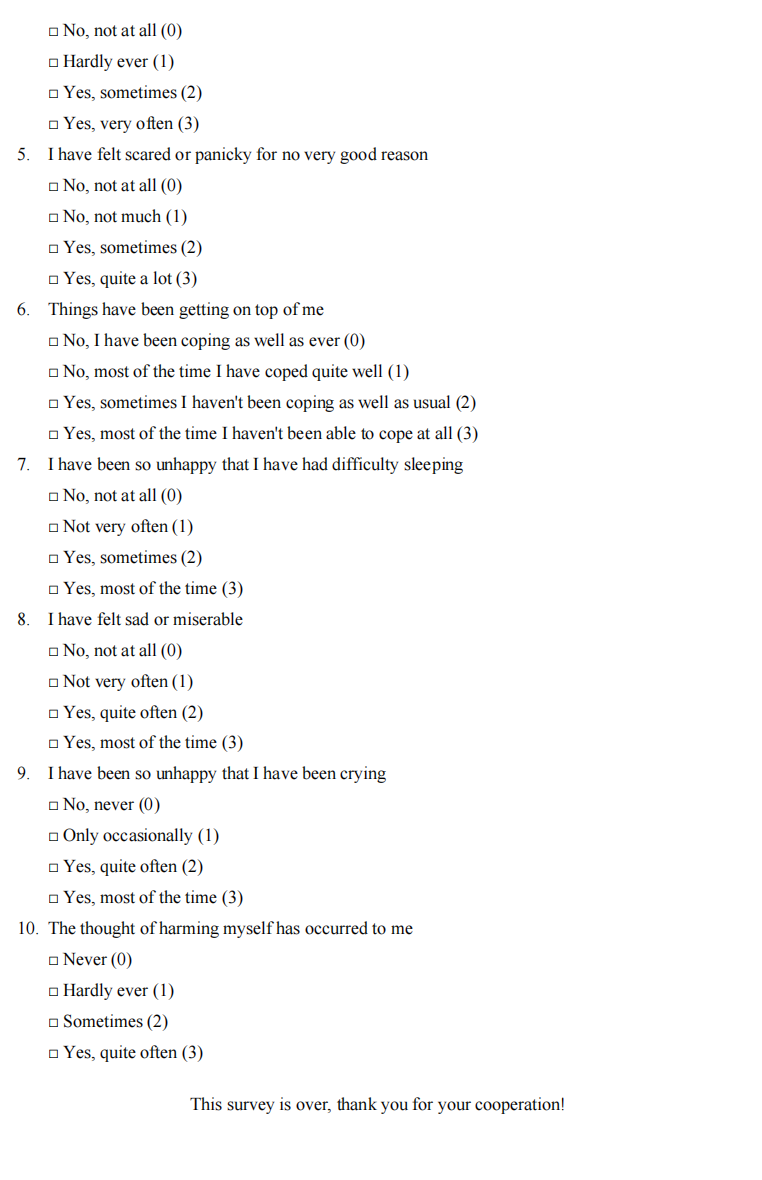


**Figure S1 Questionnaire information**

**
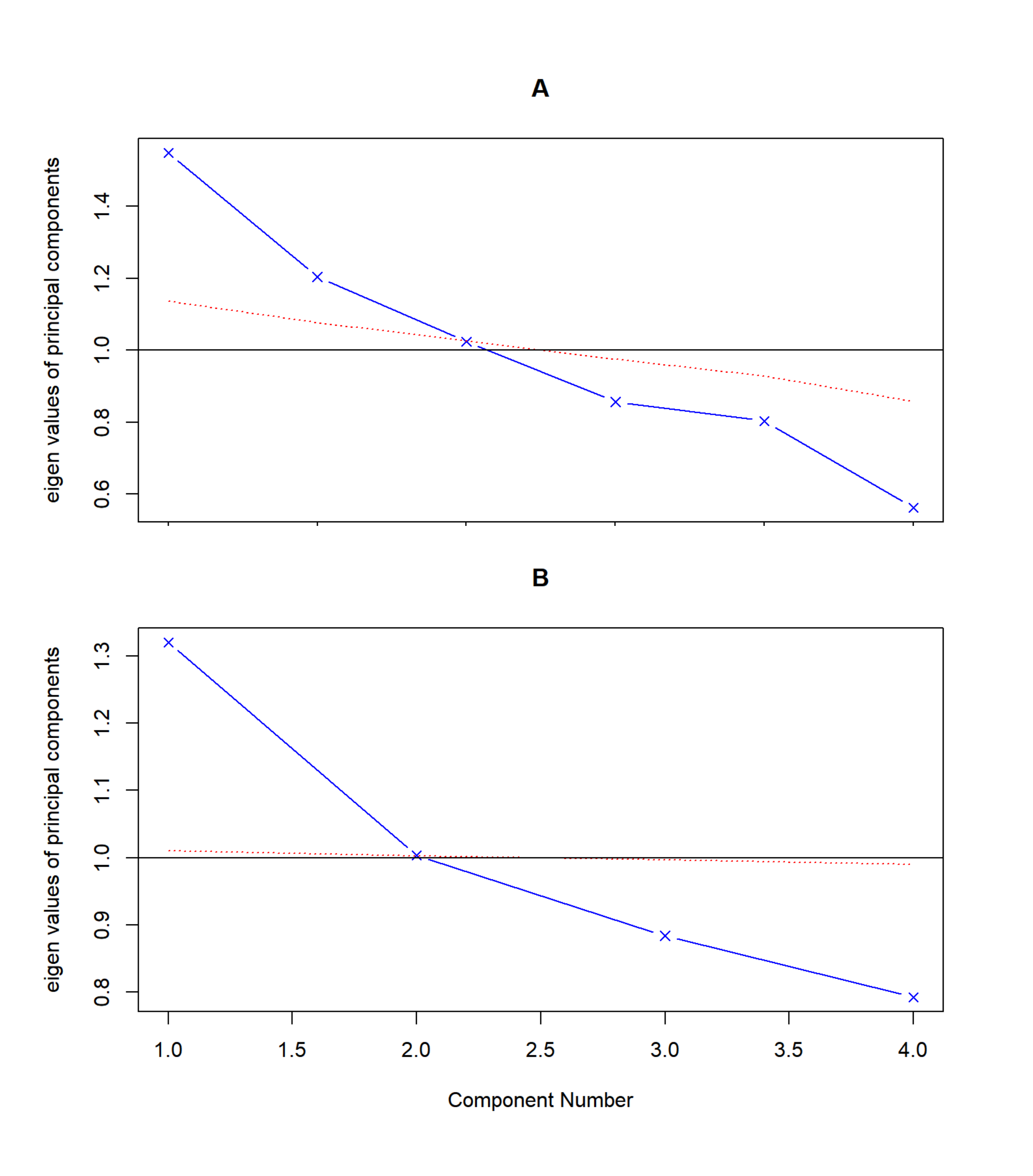
**

**Figure S2 Scree plot with parallel analysis**

Note: A. Gestational diabetes mellitus; B. Non-gestational diabetes mellitus.
